# Experiments and Practical Remarks on a Certain State of the Urine, Producing Micturitio Frequens, Dysuria, and Ischuria

**Published:** 1818-06

**Authors:** J. Wood


*For the London Medical and Physical Journal.*

### *Experiments and Practical Remarks on a certain State of the Urine, producing Micturitio Frequens, Dysuria, and Ischuria;* by J. Wood, M. D.

SEVERAL years ago (1807), I was desired to visit a gentleman, near this town, whom I found labouring under ischuria, or complete suppression of urine. He, a few days before, had frequent inclination to void his urine: then frequent inclinations, with pain and difficulty; and then ischuria. Before the latter state took place, the urine he had voided was preserved; it was nearly, I think I may say quite, as white as milk, and had been that colour from the first symptoms of his complaint. He would not suffer the catheter to be tried, which he had refused before I saw him, —and, notwithstanding every other attempt was made to relieve him, he died on the following day. Having much curiosity to know the real state of his urine, I had some of it sent into town, that I might endeavour to ascertain it.

*Exp.* 1.—Into two ounces of urine, in a wine glass, I put a few drops of a strong alcaline solution, which did not *separate* any of the parts of the urine, but evidently *dissolved* the white colouring matter in some degree, as it was less white and thinner, but still this colouring matter pervaded equally the whole contents of the glass. From this experiment I concluded, that this colouring matter was held in solution in the urine by an alkali.

*Exp.* 2.—Therefore, into another glassful of the same urine, I put a tea-spoonful of acet. distil. and the effect of this was instantaneous and surprising; for the urine immediately became clear, and of its usual colour, and a perfectly transparent glutinous matter was, at the same moment, thrown up to the surface of the urine, which was capable of being suspended on the handle of the tea-spoon, and removed from the glass: it resembled the white of an egg, but much more solid and glutinous. From this experiment I concluded that a too-alkaline state of the urine had acted on the mucus which defends the bladder; deprived the internal coat and sphincter vesicæ of their proper covering, and the latter, rendered still more sensible by every additional secretion of too-alkaline urine, had first produced micturitio frequens, then dysuria, from spasm and pain, and, lastly, ischuria.

*Exp.* 3.—Into another glass of the same urine I put some alkali, and afterwards the acet. distil., with precisely the same effect,—of separating transparent mucus to the top of the glass. I then requested my friend, and a very respectable surgeon, Mr. Fife, whose patient the gentleman was, to see me repeat the experiments; which I again did also to the pupils of Mr. Fife, with the same results.

From the above experiments, I determined to try some acid in the first case of too-alkaline urine I might meet with, —and this I did to some in the Infirmary, always with some relief. But it was not till 1812, that a very decided case, in private practice, afforded an opportunity of trying an acid in such a state of the urine. It was a lady, advanced in years (as the gentleman who died was), who suffered much from micturitio frequens, with pain, which she had had sometimes less and sometimes more. For a length of time, she had consulted several medical persons without any relief; and, at the suggestion of some female friends, whom I had attended, she desired to have my advice. On hearing what the symptoms were, and seeing some of the urine, I was led to think, from the colour and smell especially, that it was charged with uric acid, and directed magnesia and mucilage to be taken regularly; but, in a few days, this was found not to give any relief. I now discovered, that, whenever the micturitio was most frequent, the urine was then turbid, and not so at other times: I therefore desired to have some of the first turbid urine that appeared; and, by experiments similar to those related, detected mucus, held in solution by too alkaline a state of the urine. I then ordered, for the lady, nitric acid diluted with water, and covered with syrup and mucilage, in the proportion of ʒi. to. ℔i. of water, &c. and was happy to find that it afforded perfect relief in a few days, before ʒ vi. of the acid were taken, but she continued it to the extent of ʒvi. more. When the surgeon who attended this lady (and who is now dead), called on me to state the case, he said, that many things had been tried in it, and that I could not mention any thing that had not, usual in such cases,—meaning calculous complaints, in which view she had been hitherto treated. This lady continued well for four years,—when, in the spring of 1816, she had a return of the complaint, and was soon relieved by the same means; and it is remarkable, that, on the day she then sent for me, I had just seen a similar case, in a gentleman of this town, whose urine I also had examined, and who was also relieved by the nitric acid. Both occurring at the same time, and during a cold easterly wind, when the perspiration on the surface is checked, I thought it probable the secretion of the urine might be altered by this state of the weather. This gentleman had had, several years before, a severe attack of micturitio frequens, then dysuria, and ischuria; and had been relieved by the catheter in the latter state. When the sphincter is a little too sensible to the action of the urine, from a want of mucus, micturitio frequens takes place: when this sensibility is increased, by the continued action of the same causes, then occasional spasm seems to take place,—and, the causes continuing, complete spasm, or ischuria.

I should have mentioned, that the second attack the lady had was more severe than the former one, and the urine was more turbid, and strongly alkaline to the smell; and that, after the use of the nitric acid (which also improved the appetite and the strength), it became gradually clear, and of the natural colour and smell, and the painful symptoms of the complaint have gradually lessened. In this attack, the irritation was at one time so severe at the neck of the bladder, as to bring on the desire to go to stool frequently. From the change in the urine on taking the acid, may be drawn the conclusion,—that acids, taken into the stomach, do reach the urine.

I have read in the last number of the London Medical Journal, the analysis of Dr. Marcet's Essay on Calculous Disorders, and the observation of the reviewers on the subject, which have been the means of drawing this communication from my notes.

*Newcastle-upon-Tyne; April* 12, 1818.

**P. S.—I do not know that mucus was ever separated from the urine, and obtained in the state as described in my second experiment: it certainly surprised me; and chiefly by the instantaneous separation of it in that pure state described. The experiment was as fine a one as any I have ever seen.**

---

*For the London Medical and Physical Journal.*

